# Simultaneous Determination of Eight Alkaloids in Rat Plasma by UHPLC-MS/MS after Oral Administration of *Coptis deltoidea* C. Y. Cheng et Hsiao and *Coptis chinensis* Franch

**DOI:** 10.3390/molecules21070913

**Published:** 2016-07-14

**Authors:** Lu Liu, Zhi-Bin Wang, Yang Song, Jing Yang, Li-Jun Wu, Bing-You Yang, Qiu-Hong Wang, Li-Qian Wang, Ru-Xuan Wang, Chun-Juan Yang

**Affiliations:** 1 Department of Pharmaceutical Analysis and Analytical Chemistry, College of Pharmacy, Harbin Medical University, No. 157 Baojian Road, Nangang District, Harbin 150081, China; a1534064875@163.com (L.L.); wulijun_4300@hotmail.com (L.-J.W.); wangliqian93@163.com (L.-Q.W.); iwangruxuan@163.com (R.-X.W.); 2 Key Laboratory of Chinese Materia Medica (Ministry of Education), Heilongjiang University of Chinese Medicine, 24 Heping Road, Xiangfang District, Harbin 150040, China; wzbmailbox@126.com (Z.-B.W.); ybywater@163.com (B.-Y.Y.); qhwang668@sina.com (Q.-H.W.); 3 School of Pharmacy, China Medical University, Shenyang 110001, China; songyanglhyb1998@163.com; 4 Analytical Department, Johnson & Johnson, 199 Grandview Road, Skillman, NJ 08558, USA; jjsprite09@gmail.com

**Keywords:** alkaloids, UHPLC-MS/MS, method development, rat plasma

## Abstract

A ultra-high performance liquid chromatography-electrospray ionization tandem mass spectrometry (UHPLC-ESI-MS/MS) method was successfully developed and validated for the identification and determination of eight alkaloids: tetrahydropalmatine (**A**); palmatine (**B**); magnoflorine (**C**); columbamine (**D**); berberine (**E**); worenine (**F**); berberrubine (**G**) and coptisine (**H**) in rat plasma, which are the active components in *Coptis deltoidea* C. Y. cheng et Hsiao (**CCY**) and *Coptis chinensis* Franch (**CF**). The chromatographic separation of analytes was successfully achieved on an Agilent SB-C_18_ column (1.8 µm, 150 mm × 2.1 mm) using a programme with a mobile phase consisting of acetonitrile and water containing 0.3% acetic acid at a flow rate of 0.25 mL/min. The analytes were detected with a triple quadrupole tandem MS in multiple reaction monitoring (MRM) mode and an electrospray ionization (ESI) source in positive mode. The validated method showed good linearity over a wide concentration range (*r*^2^ > 0.991), and lower limits of quantification (LLOQ) less than 1.1 ng/mL for all analytes, and matrix effects ranged from 85.2% to 106.8%. The mean extraction recoveries were no less than 86.4%, and the precision and accuracy were within the acceptable limits. All analytes were proven to be stable during sample storage and analysis procedures. The method validation results demonstrated that the proposed method was sensitive, specific, and reliable, which could lay a foundation for the pharmacokinetic study of eight analytes after oral administration of **CCY** and **CF** in subsequent studies.

## 1. Introduction

*Rhizoma Coptidis* (**RC**), the dried rhizome of *Coptis deltoidea* C. Y. Cheng et Hsiao (**CCY**), *Coptis chinensis* Franch (**CF**), or *Coptis teeta* Wall, the species in the genus Coptis (family Ranunculaceae), has been used in China for thousands of years [1] and is recorded with the Chinese name of “Huang Lian” in more than thirty classical Chinese medicine books [2]. In Traditional Chinese Medicine (TCM) it could “clear the damp-heat, quench the fire, and counteract the poison” [3], while recent studies have indicated that **RC** can be used to treat hypertension and hyperlipidemia, dysentery and gastroenteritis, liver and hepatobiliary diseases, etc. [4,5] **CCY**, commonly known as “Ya Lian”, may have a lower yield due to the long growth period and other physiological characteristics, make it scarce [6]. **CF** often referred to as ”Wei Lian”, has been more widely used as the major ingredient in many Chinese herbal formulas such as oral liquid “shuang huanglian” [7]. This plant is mainly produced in the Chongqing, Hubei, Guizhou, Shanxi provinces of China. Furthermore, differences in kinds and content of alkaloid components exist between **CCY** and **CF** [8], which are caused by the differences in their growth conditions, storage, and processing of the herb.

**Figure 1:**
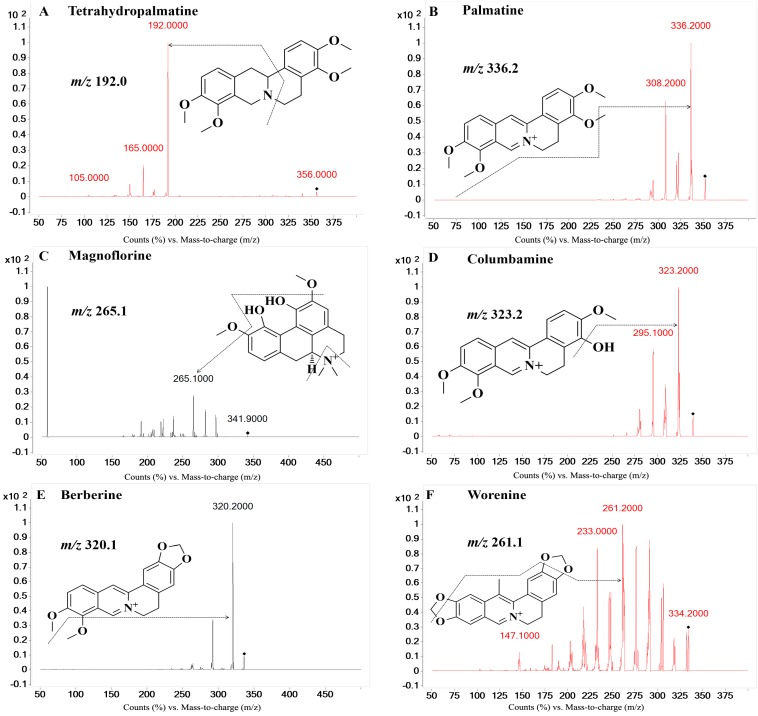

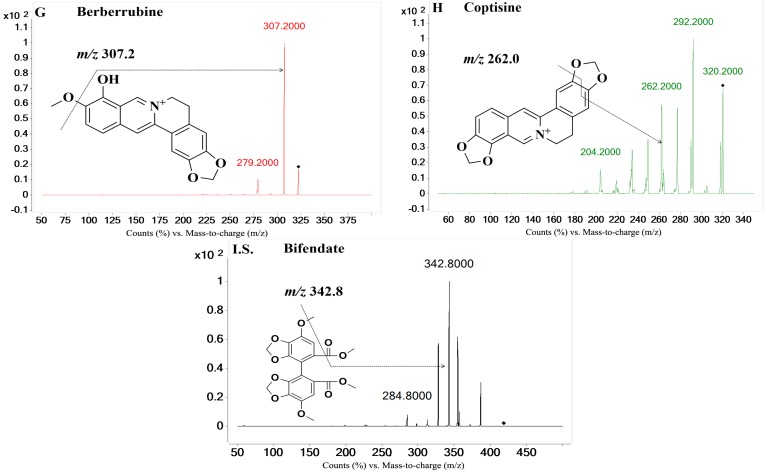
MS/MS fragmentation patterns of tetrahydropalmatine (**A**); palmatine (**B**); magnoflorine (**C**); columbamine (**D**); berberine (**E**); worenine (**F**); berberrubine (**G**); coptisine (**H**); bifendate (**I.S.**).

On the basis of previous studies, the major active constituents in **CCY** and **CF** are alkaloids, including tetrahydropalmatine (**A**); palmatine (**B**); magnoflorine (**C**); columbamine (**D**); berberine (**E**); worenine (**F**); berberrubine (**G**) and coptisine (**H**) (Figure 1), etc. In recent years, various biological activities of these components have been reported, such as antimicrobial [9], anticancer [10,11], anti-oxidative [12] and cardioprotective [13] effects, etc. For example, berberine significantly reduced myocardial ischemia/reperfusion-induced myocardial infarct size, improved cardiac function, and suppressed myocardial apoptosis and oxidative damage [14]. It is found that five major alkaloids in **RC** prevented body weight gain, reduced the serum total cholesterol, and increased the high-density lipoprotein cholesterol of hamsters [15]. As for the other analytes, coptisine, palmatine, magnoflorine and berberrubine, have been reported to have antimicrobial or anti-inflammatory properties [16,17]. Nevertheless, to the best of our knowledge, little research has addressed the pharmacokinetics of other alkaloids including magnoflorine, worenine, berberrubine from **CCY** and **CF**, which also showed a wide range of useful biological activities, such as antifungal [18], anti-inflammatory [19], and antitumor activities [20].

Earlier publications have described methods, including high-performance liquid chromatography with ultra-violet detection [21], high-performance liquid chromatography with DAD detection [22], and high-performance liquid chromatography-electrospray ionization-tandem mass spectrometry [23,24] that have been used in the qualitative or quantitative analysis of alkaloids from the genus Coptis, single herb or herbs used together in couples (Coptis-Evodia herb couple [25], scutellaria-coptis herb couple [26]), at most six or seven alkaloids simultaneously. However, to date, there are few publications concerning **CCY**, especially the pharmacokinetics. Therefore, it is of great importance to develop a specific, sensitive and simple method to simultaneously determine the main components of **CCY** and **CF** in biological samples which could be applied in compare the pharmacokinetic characteristics of **CCY** and **CF** in follow-up studies.

In this study, a rapid and sensitive high-performance liquid chromatography-electrospray ionization tandem mass spectrometry was developed for the simultaneous determination of eight alkaloids in rat plasma. The method we developed should be useful in pharmacokinetics studies after oral administration of **CCY** and **CF** which could provide important and valuable information for improving **RC** clinical therapeutic efficacy as well as a better understanding of the differences and efficacy of these two drugs. This will be the next program of our research team.

## 2. Results and Discussion

### 2.1. Method Development

#### 2.1.1. UHPLC-MS/MS Optimization

Alkaloids had stronger signal responses in the positive ion mode compared to the negative one. The MS/MS ion-transitions were monitored in MRM mode to increase the specificity of the detection method to meet the requirements of the complexity of biological samples. In the total ionization chromatography (TIC) spectra, the analytes formed predominantly protonated molecules at *m*/*z* 356.0 for tetrahydropalmatine (**A**), at *m*/*z* 342.2 for magnoflorine (C), at *m*/*z* 339.2 for columbamine (**D**), at *m*/*z* 322.2 for berberrubine (**G**), at *m*/*z* 418.9 for bifendate (**I.S.**). While, palmatine (**B**), berberine (**E**), worenine (**F**) and coptisine (**H**) predominantly formed quaternary ammonium ions [M]^+^ at 352.2, 336.2, 334.2 and 320.0 in the TIC spectra. Figure 1 shows the ion pairs of the eight analytes and the I.S. In order to make this analytical method more accurate, we used the standard of the analytes and the internal standard (I.S.) to search for the qualifier ions. To obtain the maximum sensitivity of the MRM, parameters such as fragmentor, collision energy and the nitrogen flow rate were optimized. Parameters mentioned above were listed in Table 1.

The complexity of the chemical constituents of TCM may influence the qualitative and quantitative analysis of target compounds, so we used a 15 cm Agilent SB-C_18_ column (1.8 µm, 150 mm × 2.1 mm) to avoid any crosstalk of the interfering constituents. Various ratios of organic phase (methanol, acetonitrile) to water phase inclusion of pH modifiers (ammonium acetate, formic acid, ammonia) were tested to obtain good separations and ideal peak shapes. The mobile phase consisting of a 0.3% acetic acid aqueous solution (A) and acetonitrile (B) was run at a flow rate of 0.25 mL/min according to the gradient elution program as shown in Table 2. The column temperature was set at 40 °C and the injection volume was 10 µL.

#### 2.1.2. Selection of the Internal Standard and the Extraction Method

Compared with some compounds (such as theophylline, phenacetin, etc.) we tested, the I.S. bifendate had a more suitable retention time, a good peak shape in the chromatographic separation. Extraction methods including liquid–liquid extraction (LLE) and protein precipitation with methanol, acetonitrile were tested during the sample preparation. For protein precipitation, the lower recovery and obvious matrix effect hindered further development. Moreover, among all the extraction solvents tested (ethyl acetate, diethyl ether, chloroform, acetone [27]), we found that LLE with acetone produced the highest response and negligible matrix effect.

### 2.2. Method Validation

#### 2.2.1. Selectivity and Specificity

**Figure 2:**
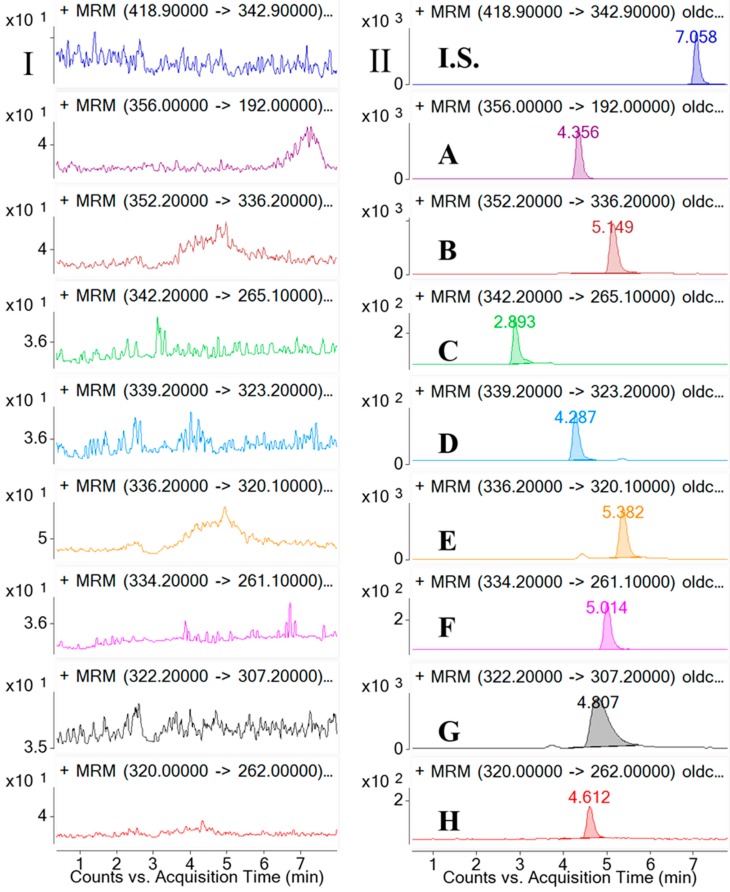

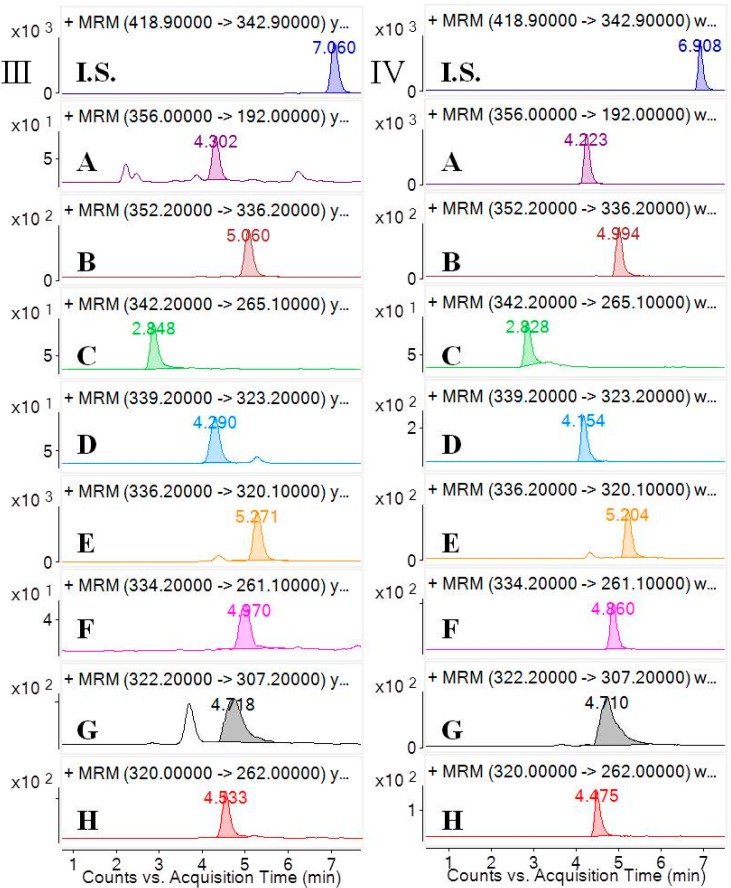
Typical MRM chromatograms of tetrahydropalmatine(**A**); palmatine (**B**); magnoflorine (**C**); columbamine (**D**); berberine (**E**); worenine (**F**); berberrubine (**G**); coptisine (**H**); bifendate (**I.S.**) in rat plasma: (**I**) Blank rat plasma; (**II**) Blank plasma spiked with eight alkaloids and I.S.; (**III**) a plasma sample from a rat 0.25 h after oral administration of *Coptis deltoidea* C. Y. cheng et Hsiao (**CCY**) extract; (**IV**) a plasma sample from a rat 0.75 h after oral administration of *Coptis chinensis* Franch (**CF**) extract.

Figure 2 shows the representative MRM chromatograms of the blank rat plasma (I); a spiked plasma sample with the analytes and the I.S. (low quality control sample, LQC, II); a plasma sample collected at 0.25 h after an oral administration of **CCY** extract (III); a plasma sample collected at 0.75 h after an oral administration of **CF** extract (III).

The results showed that there were no endogenous substance peaks from the rat plasma samples and drug metabolite peaks interfering with the analytes and the I.S. at the retention times. And in a LQC sample, compound **A**–**H** and **I.S.** eluted at 4.356, 5.149, 2.893, 4.287, 5.382, 5.014, 4.807, 4.612, 7.058 min, respectively. The analytes could be easily differentiated from the rat plasma matrix and quantitatively determined at the lower limit of quantification (LLOQ) level.

#### 2.2.2. Linearity and Lower Limit of Quantification

The calibration curve, correlation coefficient (*r*^2^), linear range, and LLOQ of each component are shown in Table 3. The lowest concentrations with RSD% < 20% were taken as LLOQs with a signal-to-noise (*S*/*N*) ratio > 10, which was sensitive enough for the pharmacokinetic studies in rat plasma. All correlation coefficients exceeded 0.9905 which indicated that eight analytes exhibited good linearity.

#### 2.2.3. Precision and Accuracy

The intra- and inter-day precision and accuracy of the method are presented in Table 4 which was evaluated by measuring six replicates of LLOQ and quality control sample (QC) samples at three concentration levels (LQC, medium quality control sample, MQC, and high quality control sample, HQC) on the same day for three consecutive days. The intra and inter-day precisions (RSD%) of these analytes were all less than 14.4% for QC samples and 18.8% for LLOQ, respectively, and the accuracy ranged from −14.4% to 14.1%. The results indicated that the precision and accuracy values were acceptable.

#### 2.2.4. Extraction Recovery and Matrix Effects

The mean recovery and matrix effect data were evaluated by analyzing QC samples at three concentrations levels (LQC, MQC, and HQC) with six replicates. The data on the extraction recovery and the matrix effects of eight alkaloids determined in the plasma are depicted in Table 5. Mean recoveries of eight analytes of the QC samples were in the range of 86.4%–100.0%, and the mean recovery of the I.S. was 88.7% ± 13.7%. The matrix effects derived from the QC samples ranged from 85.2% to 106.8%, and for the I.S., it was 98.2% ± 11.1%. Thus, it was confirmed that LLE with acetone was a feasible and appropriate method for the extraction of the analytes and the I.S., and moreover, the method was reliable and little matrix effect occurred.

#### 2.2.5. Stability

Freeze-thaw stability, short-term stability, long-term stability and post-preparative stability were determined by measuring the response area ratio (analyte/I.S.) of stability samples against freshly prepared QC samples with identical concentrations. Summary of the stability data of the eight analytes under different conditions are shown in Table 6. The measured concentrations for the eight alkaloids at each QC levels deviated within 14.9%, which indicated that the method is applicable for routine analysis.

### 2.3. Method Comparison with Existing Reports

**Figure 3:**
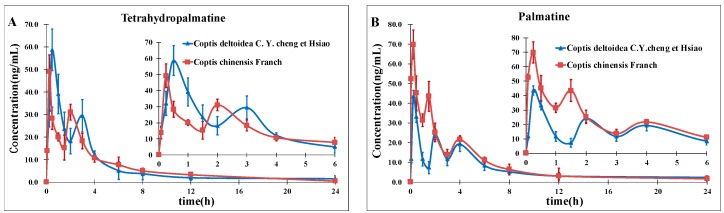

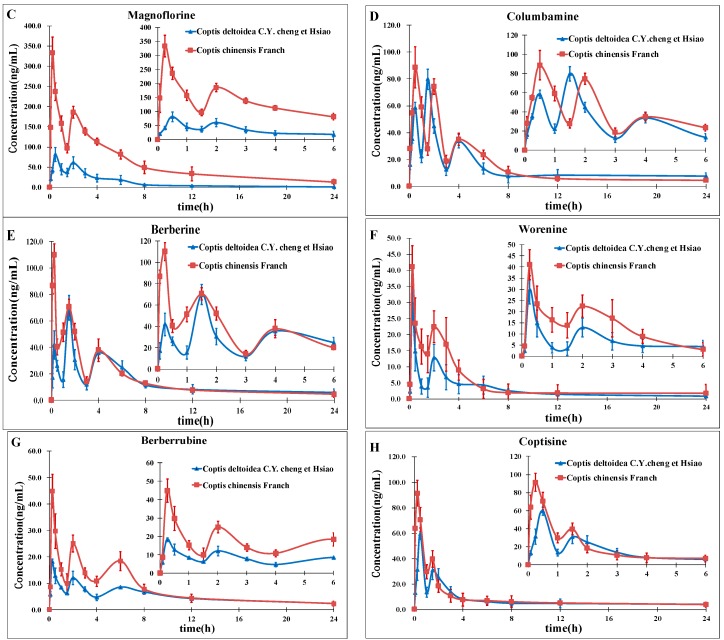
Mean concentration-time profiles of tetrahydropalmatine (**A**); palmatine (**B**); magnoflorine (**C**); columbamine (**D**); berberine (**E**); worenine (**F**); berberrubine (**G**); coptisine (**H**) in rat plasma after oral administration of **CCY** and cf. Each point represents the mean ± SD (*n* = 14).

There are several reports on assaying berberine, coptisine, palmatine in *Rhizome Coptidis* [28,29,30], but no analytical method was developed for the simultaneous determination of the given eight alkaloids in this study. Most studies mainly focused on the determination of several kinds of alkaloids present in high contents. There was a report on the simultaneous determination of coptisine, berberine, palmatine, magnoflorine and columbamine from wine-processed *Rhizoma Coptidis* in rat plasma [8]. The LLOQs were 0.5 ng/mL for coptisine and columbamine, 2.03 ng/mL for berberine, 2.2 ng/mL for palmatine, and 5.5 ng/mL for magnoflorine. The LLOQ in this study was 0.1 ng/mL for palmatine and berberine, 0.2 ng/mL for coptisine, 0.5 ng/mL for columbamine, tetrahydropalmatine and worenine and 1.1 ng/mL for berberrubine and magnoflorine. The chromatographic run time was 9 min. In comparison, the method described in this study is much shorter and more suitable for clinical applications. *Rhizoma Coptidis* is a main herb in traditional Chinese medicine prescriptions such as Xilixiao capsule, Shensong yangxin capsule and Yiqing Capsule which has several species and explicit boundaries. To improve the understanding of the difference between pharmacokinetics and even pharmacology of these crude herbs, a selective and sensitive analytical method for the simultaneous quantification of the main bioactive components in this herb after oral administration of TCM in plasma is required. To the best of our knowledge, this is the first time tetrahydropalmatine, palmatine, magnoflorine, columbamine, berberine, worenine, berberrubine and coptisine has been simultaneously determined using an LC-MS-MS method. Furthermore, Figure 3 shows the mean plasma concentration-time profiles of the eight alkaloids: tetrahydropalmatine (**A**); palmatine (**B**); magnoflorine (**C**); columbamine (**D**); berberine (**E**); worenine (**F**); berberrubine (**G**); coptisine (**H**) in rat plasma after oral administration of **CCY** and cf. The information described above might be helpful for further studies on the pharmacokinetics of **CCY** and **CF** and beneficial for application of these Traditional Chinese Medicines in clinical therapy. Moreover, it has the potential to provide valuable contributions to the field of plant toxin analyses for toxicity studies.

## 3. Experimental Section

### 3.1. Material and Regents

The eight reference standards (**A**–**H**, purity 98.0%) were all obtained from the Chengdu Pufei De Biotech Co., Ltd. (Chengdu, Sichuan, China). The I.S. (bifendate) with a purity of 99.9% was purchased from the National Institutes for Food and Drug Control (Beijing, China). Water (H_2_O) was purified by a Milli-Q system (Millipore, Billerica, MA, USA) in our laboratory. HPLC-grade methanol and acetonitrile were purchased from Amethyst Chemicals (Beijing, China). HPLC-grade acetic acid was purchased from CNW Technologies (Düsseldorf, Germany). All other reagents were analytical-grade.

**CF** was the dried rhizome of the *Coptis chinensis* Franch which was purchased from Bozhou Medicinal Material Company (Anhui, China) and authenticated by Professor Zhenyue Wang in Heilongjiang University of Chinese Medicine. **CCY** was the dried rhizome of the *Coptis deltoidea* C. Y. cheng et Hsiao which was collected from Hongya County, Meishan City, Sichuan Province, China, which is protected as a vulnerable plant in China [31], and also authenticated by Professor Zhenyue Wang.

### 3.2. Preparation of ***CCY*** Extract and ***CF*** Extract

**CCY** and **CF** were crushed to pieces before use. A dried powder sample of each (100 g) was extracted three times (1 h each time) under reflux with 70% ethanol at a material:liquid ratio of 1:10 and finally the extracted solution was concentrated. The residue was reconstituted in water to obtain a concentration equivalent to 0.285 g/mL of the **CCY** extract and 0.345 g/mL of the **CF** extract. The eight analytes′ contents in both extracts were measured quantitatively by HPLC-DAD. The contents of **A**–**H** in **CCY** extract were 0.046, 0.640, 0.269, 0.865, 1.637, 0.022, 0.230 and 1.073 mg/mL, respectively. Meanwhile, the contents of **A**–**H** in **CF** extract were 0.013, 0.840, 0.475, 1.598, 1.868, 0.038, 1.254 mg/mL and 1.766 mg/mL, respectively.

### 3.3. Instrumental and Chromatographic Conditions

The UHPLC-MS/MS system consisted of an Agilent series 1290 system (Agilent, Santa Clara, CA, USA) connected online to a 6430 triple-quadrupole mass spectrometer with an electrospray ionization (ESI) interface (Agilent). Chromatographic separation was performed on an Agilent SB-C_18_ column (1.8 µm, 150 mm × 2.1 mm; Agilent) at a flow rate of 0.25 mL/min. The mobile phases were 0.3% acetic acid aqueous solution (A) and acetonitrile (B) with gradient elution program listed in Table 2. The injection volume was 10 µL and the column temperature was 40 °C. Eight analytes and the I.S. were monitored using multiple reaction monitoring (MRM) mode in the positive ionization mode with the following settings after optimization: source voltage of 4.0 kV, drying gas flow rate of 11 L/min, nebulizer pressure of 20 psi, source temperature (TEM) of 300 °C. Gases were 99.999% nitrogen. The MRM transitions, qualifier ions and other MS parameters were listed in Table 1.

### 3.4. Preparation of Calibration Standards and Quality Control Samples

Compounds **A**–**H** were accurately weighed and dissolved in methanol to make up the stock solutions (1014, 107, 216, 223, 108, 222, 221, 100 µg/mL) which were kept at −4 °C when not in use. A 2 µg/mL solution for the I.S. was also prepared in methanol. The mixed standard stock solution were prepared by dilutions of the stock solution with methanol to obtain a final mixed standard solution containing 2028 ng/mL of **A**, 428 ng/mL of **B**, 4320 ng/mL of **C**, 2230 ng/mL of **D**, 442 ng/mL of **E**, 2220 ng/mL of **F**, 4420 ng/mL of **G**, 800 ng/mL of **H**, respectively. And then further diluted in pattern of 1:4:2.5:4:2.5:4:2.5 to produce the working solutions with a series of concentrations. Calibration standards were prepared by spiking appropriate amounts of the mixture standard working solutions into the blank rat plasma to give the nominal concentration range of 0.5–2028 ng/mL for **A**, 0.1–428 ng/mL for **B**, 1.1–4320 ng/mL for **C**, 0.6–2230 ng/mL for **D**, 0.1–442.0 ng/mL for **E**, 0.6–2220 ng/mL for **F**, 1.1–4420 ng/mL for **G**, 0.2–800 ng/mL for **H**, respectively. Three levels of the QC samples (2.0, 50.7 and 1622 ng/mL for **A**, 0.4, 10.7 and 342 ng/mL for **B**, 4.3, 108 and 3456 ng/mL for **C**, 2.2, 55.8 and 1784 ng/mL for **D**, 0.4, 11.1 and 354 ng/mL for **E**, 2.2, 55.5 and 1776 ng/mL for **F**, 4.4, 111 and 3536 ng/mL for **G**, 0.8, 20.0 and 640 ng/mL for **H**) in drug-free plasma were prepared in the same way as the calibration standards.

### 3.5. Animals and Sample Preparation

Blood samples from male Wistar rats which were divided into two groups (body weight 200 ± 15 g) were obtained at specific time points after the oral administration of **CCY** (0.148 g/kg) and **CF** (0.179 g/kg), which is equal to the dosage of original herbs of 0.09 g per rat (220 g body weight) [32]. The rats were supplied by Center for Drug Safety Evaluation of Heilongjiang University of Chinese Medicine (Harbin, Heilongjiang, China) and bred under standard laboratory conditions (temperature, 21 ± 1 °C; relative humidity, 60% ± 5%) with free access to chow and water. All rats were fasted overnight with free water supply before experiments, and they had access to water during the experiment. All experimental procedures conducted according to the European Community guidelines for the use of experimental animals [33]. We have received an animal ethical approval before our experimentation from animal ethics committee of Harbin Medical University. The title of the animal ethical approval was “The pharmacokinetics study of *Rhizoma Coptidis*” and this study was approved by the ethics committee of Harbin Medical University on 15 December 2015. For each animal, after the oral administration of **CCY** and **CF** extracts, 0.25 mL blood samples were withdrawn from the retinal venous plexus into heparinized tubes at several time points. The rat blood samples were processed to obtain plasma by centrifugation at 12,000 rpm for 10 min at −4 °C. 100 µL plasma sample and 50 µL I.S. were transferred to a 10 mL glass tube and vortexed for 60 s, and extracted with 3 mL acetone vortex-mixing for 120 s. After centrifugation at 3800 rpm for 5 min, the upper organic layer was transferred to another tube and evaporated to dryness under a gentle stream of nitrogen at 40 °C. The residue was reconstituted with 100 µL of the mobile phase, vortexed for 60 s and filtered by a 0.22 µm membrane. A 10 µL aliquot of the solution was injected into the LC-ESI-MS/MS system for analysis.

### 3.6. Method Validation

#### 3.6.1. Selectivity and Specificity

Selectivity was investigated by analyzing blank samples from fourteen different batches of rat plasma. The representative MRM chromatograms of blank plasma and corresponding blank plasma spiked with the eight analytes and the I.S., and the plasma samples from the rats after oral administration of the **CCY** and **CF** extract were compared. The selectivity of these analytes are shown in Figure 2. Specificity refers to the ability of analytical method to differentiate and quantify the analytes in the presence of other components.

#### 3.6.2. Linearity and Lower Limit of Quantification

The calibration curves were determined by plotting the peak area ratio (Y) of the analytes to the I.S. versus the nominal concentration (X) of analytes with weighted (1/X^2^) least square linear regression. The LLOQ for analytes of the assay was defined as the lowest concentrations of the calibration curve that could be quantitated with the *S*/*N* of at least 10 with sufficient precision (±20%) and accuracy (80%–120%).

#### 3.6.3. Precision and Accuracy

Intra- and inter-day precision and accuracy were investigated by determining LLOQ and QC samples at three concentration levels (six replicates for each concentration level) on three consecutive days. The concentrations of the QC samples were determined from the standard calibration curve and were analysed on the same day. Precision was expressed as the relative standard deviation (RSD%) and the accuracy was defined as the percentage relative error (RE%), both required to be within ±15% except at the LLOQ where ≤20% was acceptable.

#### 3.6.4. Recovery and Matrix Effect

The extraction recoveries of the eight analytes were assessed by comparing the mean peak areas of blank matrix samples spiked before and after extraction of the three QC samples (*n* = 6). The matrix effects of the eight analytes were assessed by comparing the mean peak areas of QC samples spiked after sample preparation with the mean peak areas of the unextracted samples (*n* = 6).

#### 3.6.5. Stability

The stability was investigated by assessing six replicates of the QC samples at three concentration levels in different conditions. These conditions included freeze-thaw stability (frozen at −20 °C and thaw cycles), room temperature stability (storage for 4 hours at ambient temperature), long term stability (storage for 2 weeks at −20 °C), and post-preparation stability (storage for 12 h after sample preparation at 4 °C). The samples were considered stable if the RE% was within 15% of the actual value.

## 4. Conclusions

A rapid, sensitive and simple LC-MS-MS method for the simultaneous determination of tetrahydropalmatine, palmatine, magnoflorine, columbamine, berberine, worenine, berberrubine and coptisine in *Coptis deltoidea* C. Y. Cheng et Hsiao and *Coptis chinensis* Franch in rat plasma was developed and validated. The high sensitivity made the analytical procedure suitable for the determination of these low concentration alkaloids in rat plasma. The method could be used for the pharmacokinetic studies and may contribute to revealing the mechanism of action and guide the clinical application of *Coptis deltoidea* C. Y. Cheng et Hsiao and *Coptis chinensis* Franch.

## Figures and Tables

**Table 1:** Quantitative, Qualifier ions and MS parameters of eight alkaloids and I.S.

Compounds	Ion Pair (*m*/*z*)	Qualifier Ion (*m*/*z*)	Fragmentor (V)	Collision Energy (V)	Polarity ^1^
Bifendate (I.S.)	418.9→342.8	284.8	78	18	Positive
Tetrahydropalmatine	356.0→192.0	165.0	159	27	Positive
Palmatine	352.2→336.2	308.2	158	30	Positive
Magnoflorine	342.2→265.1	58.2	134	22	Positive
Columbamine	339.2→323.2	295.1	160	29	Positive
Berberine	336.2→320.1	292.2	136	30	Positive
Worenine	334.2→261.1	233.0	181	49	Positive
Berberrubine	322.2→307.2	279.2	160	29	Positive
Coptisine	320.0→262.0	292.2	167	29	Positive

^1^ The cell accelerator voltage valued of these eight compounds are 5 V.

**Table 2:** Gradient elution program of mobile phase.

Time (min)	A%	B%
0	65	35
0–2	45	55
2–3	38	62
3–4.5	38	62
4.5–7.0	30	70
7.0–7.5	65	35

**Table 3:** The regression equations, linear ranges and LLOQs for the determination of the analytes in rat plasma.

Compounds	Regression Equation	*R*^2^	Linear Range (ng/mL)	LLOQ (ng/mL)
Tetrahydropalmatine	Y = 15.358X + 1.720 × 10^−2^	0.9964	0.5–2028	0.5
Palmatine	Y = 112.57X + 5.3976 × 10^−2^	0.9928	0.1–428	0.1
Magnoflorine	Y = 0.3350X + 3.272 × 10^−3^	0.9906	1.1–4320	1.1
Columbamine	Y = 2.3498X + 5.334 × 10^−2^	0.9956	0.6–2230	0.6
Berberine	Y = 18.189X + 0.117 × 10^−1^	0.9985	0.1–422	0.1
Worenine	Y = 2.4691X + 3.378 × 10^−3^	0.9907	0.6–2220	0.6
Berberrubine	Y = 12.738X + 1.730 × 10^−3^	0.9945	1.1–4420	1.1
Coptisine	Y = 2.5543X + 3.665 × 10^−2^	0.9905	0.2–800	0.2

**Table 4:** Precision and accuracy of the determination of eight alkaloids in rat plasma (*n* = 18, 6 replicates per day for 3 days).

Compounds	Spiked Concentration (ng/mL)	Measured CONC (ng/mL)	Accuracy (%)	Intra-Day Precision (%)	Inter-Day Precision (%)
Tetrahydropalmatine	0.5	0.5 ± 0.1	−1.0	17.6	18.8
	2.0	2.1 ± 0.3	−3.4	13.6	3.7
	50.7	53.1 ± 6.9	−3.0	12.9	12.9
	1622	1759 ± 172	8.4	9.1	13.8
Palmatine	0.1	0.1 ± 0.02	8.5	18.5	15.6
	0.4	0.5 ± 0.07	11.2	13.4	11.9
	10.7	11.1 ± 1.5	3.4	14.2	10.3
	342	368 ± 41.8	7.5	11.8	6.7
Magnoflorine	1.1	1.3 ± 0.1	14.1	11.7	6.6
	4.3	4.3 ± 1.1	7.1	14.3	4.7
	108	116 ± 13.8	7.5	12.0	10.3
	3456	3817 ± 485	10.4	12.5	14.4
Columbamine	0.6	0.6 ± 0.04	−14.4	14.9	14.2
	2.2	2.5 ± 0.3	9.8	14.3	3.5
	55.8	53.9 ± 6.7	−3.4	12.3	13.5
	1784	1713 ± 193	−4.0	11.0	13.4
Berberine	0.1	0.1 ± 0.01	−2.9	10.0	17.8
	0.4	0.5 ± 0.1	5.5	14.3	6.3
	11.1	10.7 ± 1.0	−7.3	8.7	12.4
	354	317 ± 30.7	−10.4	10.0	6.8
Worenine	0.6	0.6 ± 0.1	−0.1	16.8	13.9
	2.2	2.2 ± 0.3	−4.3	14.0	10.3
	55.5	51.3 ± 6.1	−7.4	12.0	12.0
	1776	1715 ± 90.6	−3.4	4.9	7.6
Berberrubine	1.1	1.1 ± 1.1	0.8	14.3	9.2
	4.4	5.3 ± 0.6	14.1	11.6	8.4
	111	112 ± 14.0	1.5	13.1	6.2
	3536	3949 ± 437	11.7	11.3	9.4
Coptisine	0.2	0.2 ± 0.03	13.4	13.8	1.8
	0.8	0.8 ± 0.1	2.1	12.7	9.9
	20.0	22.4 ± 2.6	11.8	11.9	9.0
	640	555 ± 70.3	−13.3	13.1	8.4

**Table 5:** Matrix effects and extraction recovery for the analytes in rat plasma (*n* = 6).

Compounds	Spiked Concentration (ng/mL)	Matrix Effect		Extraction Recovery	
		Mean (%)	RSD (%)	Mean (%)	RSD (%)
Tetrahydropalmatine	2.0	105.1	8.4	94.8	14.2
	50.7	100.5	8.2	95.9	7.8
	1622	104.4	7.2	94.7	4.2
Palmatine	0.4	101.7	6.2	93.1	13.6
	10.7	93.7	11.2	86.4	5.1
	342	101.8	12.5	95.6	3.0
Magnoflorine	4.3	99.1	11.4	93.4	5.5
	108	85.2	13.1	90.0	4.1
	3456	98.5	13.4	86.4	14.3
Columbamine	2.2	100.0	13.5	100.0	6.2
	55.8	98.8	5.9	90.8	8.9
	1784	101.2	9.6	98.4	12.0
Berberine	0.4	101.8	12.3	92.3	8.2
	11.1	100.5	9.3	93.2	4.0
	354	102.3	5.0	97.6	11.1
Worenine	2.2	92.5	7.8	98.9	12.1
	55.5	87.7	10.0	88.0	9.8
	1776	105.6	5.1	93.4	1.8
Berberrubine	4.4	94.7	2.4	90.6	10.2
	111	98.0	11.3	91.2	2.6
	3536	100.8	4.3	93.4	11.5
Coptisine	0.8	106.8	3.9	92.3	7.4
	20.0	99.5	5.7	92.2	4.2
	640	89.1	5.1	89.0	8.4
I.S.	2000	98.2	11.1	88.7	13.7

**Table 6:** Stabilities of the analytes in rat plasma (*n* = 6).

Compounds	Spiked Concentration (ng/mL)	Stability (% RE)			
		Freeze-Thaw	Short Term	Long Term	Post Preparative
Tetrahydropalmatine	2.0	−10.9	13.3	13.6	2.8
	50.7	−13.8	12.5	13.5	−0.8
	1622	6.2	8.0	14.5	−5.5
Palmatine	0.4	−0.2	14.9	5.9	8.5
	10.7	−2.3	−9.6	−11.2	−12.4
	342	−2.8	−1.7	12.9	−3.0
Magnoflorine	4.3	12.2	10.6	9.4	10.7
	108	−3.4	14.1	13.6	−1.0
	3456	13.3	7.4	13.2	6.7
Columbamine	2.2	12.3	11.9	11.7	6.9
	55.8	−10.2	−0.3	−2.5	−11.2
	1784	1.9	−4.2	−5.9	−1.8
Berberine	0.4	9.4	1.2	2.4	2.9
	11.1	−12.5	−1.0	−14.6	2.6
	354	−12.5	−4.1	−11.0	−4.2
Worenine	2.2	5.6	−12.9	−7.8	12.3
	55.5	4.7	8.9	9.9	5.6
	1776	−5.4	−9.8	−4.3	−6.6
Berberrubine	4.4	8.1	8.2	8.0	13.9
	111	11.3	0.01	−2.5	4.4
	3536	6.9	8.6	−0.6	11.2
Coptisine	0.8	13.1	−14.4	8.7	5.1
	20.0	2.1	−6.1	−5.6	1.8
	640	8.4	−4.8	0.6	12.2
